# Mental health interventions for individuals with serious mental illness in the criminal legal system: a systematic review

**DOI:** 10.1186/s12888-024-05612-7

**Published:** 2024-03-12

**Authors:** Maji Hailemariam, Tatiana E. Bustos, Barrett Wallace Montgomery, Garrett Brown, Gashaye Tefera, Rosemary Adaji, Brandon Taylor, Hiywote Eshetu, Clara Barajas, Rolando Barajas, Vanessa Najjar, Donovan Dennis, Jasmiyne Hudson, Julia W. Felton, Jennifer E. Johnson

**Affiliations:** 1 Charles Stewart Mott Department of Public Health, College of Human Medicine, Michigan State University, Flint, MI USA; 2 RTI International, Research Triangle Park, NC USA; 3 College of Social Work, Florida State University, Tallahassee, USA; 4 Department of Epidemiology and Biostatistics, Michigan State University, Flint, MI USA; 5 Dornsife School of Public Health, Health Management and Policy Department, Drexel University, Philadelphia, USA; 6 Georgetown University School of Medicine, Washington, DC USA; 7 College of Osteopathic Medicine, Michigan State University, Flint, MI USA; 8 College of Human Medicine, Michigan State University, Flint, MI USA; 9 Center for Health Policy & Health Services Research, Henry Ford Health, Detroit, MI USA

**Keywords:** Serious mental illness, Interventions, Criminal legal settings, Barriers, Facilitators

## Abstract

**Background:**

Globally, individuals with mental illness get in contact with the law at a greater rate than the general population. The goal of this review was to identify and describe: (1) effectiveness of mental health interventions for individuals with serious mental illness (SMI) who have criminal legal involvement; (2) additional outcomes targeted by these interventions; (3) settings/contexts where interventions were delivered; and (4) barriers and facilitating factors for implementing these interventions.

**Methods:**

A systematic review was conducted to summarize the mental health treatment literature for individuals with serious mental illness with criminal legal involvement (i.e., bipolar disorder, schizophrenia, major depressive disorder). Searches were conducted using PsychINFO, Embase, ProQuest, PubMed, and Web of Science. Articles were eligible if they were intervention studies among criminal legal involved populations with a mental health primary outcome and provided description of the intervention.

**Results:**

A total of 13 eligible studies were identified. Tested interventions were categorized as cognitive/behavioral, community-based, interpersonal (IPT), psychoeducational, or court-based. Studies that used IPT-based interventions reported clinically significant improvements in mental health symptoms and were also feasible and acceptable. Other interventions demonstrated positive trends favoring the mental health outcomes but did not show statistically and clinically significant changes. All studies reported treatment outcomes, with only 8 studies reporting both treatment and implementation outcomes.

**Conclusion:**

Our findings highlight a need for more mental health research in this population. Studies with randomized design, larger sample size and studies that utilize non-clinicians are needed.

**Supplementary Information:**

The online version contains supplementary material available at 10.1186/s12888-024-05612-7.

## Background

Globally, individuals with mental illness get involved with the criminal legal system at a greater rate than the general population [1, 2]. For example, there are more people with mental illness currently in the U.S. criminal legal system than those receiving care in inpatient psychiatric hospitals [3]. Individuals with mental illness have an elevated risk of criminal legal involvement including interacting with 911 calls, local law enforcement, pretrial jail detention, court appearances, specialty courts, jail sentences, probation, and parole [2, 4]. Once involved in the criminal legal system, individuals with mental illness are also more likely than those without mental illness to have multiple incarcerations, serve longer sentences, be denied probation or parole supervision, have their probation or parole revoked for technical violation, and return to jail in the first year after release [5–7].

Given the large degree of overlap of criminal legal involvement of individuals with mental illness, the criminal legal system serves as a *de facto* public health system in most high-income countries [8]. On the contrary, mental health care needs of criminal legal involved individuals in low-and middle-income countries (LMICs) are often neglected and underfunded [9]. Criminal legal settings in LMICs also experience a higher burden of mental health and substance use disorders compared to high-income countries [10, 11]. Moreover, globally, criminal legal settings are complex systems that face significant barriers to implementing programs and interventions due to limited institutional capacity and resources, lack of qualified workforce, restrictive policies, lack of programmatic support, and navigating varied treatment preferences and staff attitudes [12]. These inherent institutional barriers create more complexities to the undertaking of delivering mental health interventions in the criminal legal system [13].

Evidence from most high-income countries indicate that many individuals with mental illness who report criminal legal involvement unfortunately have their first contact with a mental health service provider while in a correctional facility [14–16]. Many correctional facilities also have distinct mental health units [17]. However, a significant proportion of individuals with criminal legal involvement, particularly those in LMICs, still face barriers to accessing mental health care both during incarceration and in the community [18]. Correctional facilities often struggle to meet the treatment needs of individuals with mental illness in their custody [12, 19].

In recent years, there has been interest in integrating mental health interventions with community reentry efforts to improve health and criminal legal outcomes [20–22]. This effort also includes diversion programs, alternatives to incarceration, and better community-based mental health crisis services to keep people with serious mental illness out of jail [22]. For individuals with serious mental illness leaving jails and prisons, successful community reentry involves prompt linkage to community mental health, medical care and substance use services. Community-based alternatives to incarceration including jail diversion, mental health courts, reentry programs, crisis interventions and other programs have been introduced to improve the mental health and criminal legal outcomes of individuals with mental health needs [21].

Community-based and correctional or healthcare facility-based interventions have been introduced to address the needs of individuals with mental health challenges in the criminal legal system [9, 23]. For example, courts started requiring routine mental health screening and treatment for populations with criminal legal involvement [24]. Various evidence-based interventions are being implemented with the aim of reducing the number of individuals with mental illness in correctional facilities [21].

The Sequential Intercept Model (SIM) helps to understand the various points at which individuals with SMI come into contact with the criminal legal system. The model provides six criminal legal intercepts ranging from communities to community corrections where interventions are possible to implement to prevent involvement with the criminal legal system.

Along these intercepts, jail diversion programs, mental health courts, critical time intervention and other programs have been designed to improve criminal legal involvement and public health outcomes for individuals with mental health needs who have criminal legal involvement. However, several barriers to implementation of evidence-based interventions with criminal legal-involved populations persist [25]. Moreover, prior systematic reviews have focused on program impacts on substance use and return to prison outcomes, demonstrating limited evidence synthesizing the extent of improvements made primarily on individual mental health outcomes.

## Objectives

This systematic review summarizes the mental health treatment literature for individuals with serious mental illness who have criminal legal involvement. We define serious mental illness (SMI) as psychotic and affective disorders associated with long-term and persistent disability and substantial functional impairment. These include schizophrenia, bipolar disorder or major depressive disorder. We define the mental health treatment literature for individuals with SMI as the peer reviewed scientific literature on the mental health interventions in this population, in which measures of mental health are the primary outcome. Given the emphasis in other systematic reviews, the current project builds the evidence base on how mental health interventions affect individual mental health outcomes among individuals with serious mental illness who have criminal legal involvement. Specifically, the review aimed to identify and describe: (1) mental health interventions for individuals with SMI who have criminal legal involvement; (2) the mental health outcomes used, (3) additional outcomes targeted by these interventions; (4) settings/contexts where interventions were delivered; and (5) barriers and facilitating factors for implementing the interventions using the Consolidated Framework for Implementation Research (CFIR). The CFIR is a conceptual framework that helps to map out implementation contexts and identify potential determinants for the implementation and effectiveness of an intervention. The framework has five domains including intervention characteristics, outer setting, inner setting, characteristics of individuals, and process of implementation.

## Methods

We used the Preferred Reporting Items for Systematic Reviews and Meta-Analysis (PRISMA) guideline to structure this review. Methods of data collection and analysis were documented *a priori*. This systematic review protocol was registered in the PROSPERO registration system on 06/07/2020 under registration number CRD42020172627. An update to the information provided in PROSPERO was done to reflect changes in the study.

### Eligibility criteria

Eligible studies included articles that (1) were peer reviewed; (2) were written in English; (3) examined a specific intervention among individuals with SMI who have criminal legal involvement; (4) had mental health as a primary outcome; and (5) provided a description of the intervention used. Articles were excluded if they (1) were a review of or secondary analysis of the work of others; (2) lacked original data relating to intervention outcomes; or (3) were not peer reviewed. We did not exclude studies based on study design.

### Information sources

We searched electronic databases to identify potentially eligible studies. These include PsychINFO (1887–2020), PubMed (1946–2020), Embase (1976–2020), Web of science (1900–2020), ProQuest: ERIC and CSA Social Services Abstract, and Medline (PubMed) (1806–2020). There were no restrictions to time in order to find the most comprehensive set of articles focused on mental health interventions among criminal involved populations with SMI. Additional articles were identified by reviewing reference lists of eligible papers. We conducted an initial search in June 2020, and additional time-restricted searches (June 2020 – June 2022) were run to identify more recently published studies.

### Search strategy

We used different combinations of pre-identified keywords. All search terms were identified from a preliminary overview of literature focused on mental health interventions in criminal legal settings. The keywords used included: “evidence-based,” “mental health,” ”EBIs,””interventions,” “mental illness,” “justice-involved,” “jail,” “prison,” “services,” “inmates,” “serious mental illness,” “depression,” “schizophrenia”, “bipolar disorder”, “prisoners,” “behavioral health,” “probation,” “court diversion,” “criminal,” “diversion,” and “parole”. We combined these terms using “AND” or “OR”. If available, searches utilized a filter to only identify “peer-reviewed” articles written in the English language. Otherwise, we filtered out articles in other languages and papers that did not go through a peer-review process manually. Duplicate articles were removed once all articles from these initial searches were identified and stored in a file.

### Study selection

**Fig. 1:**
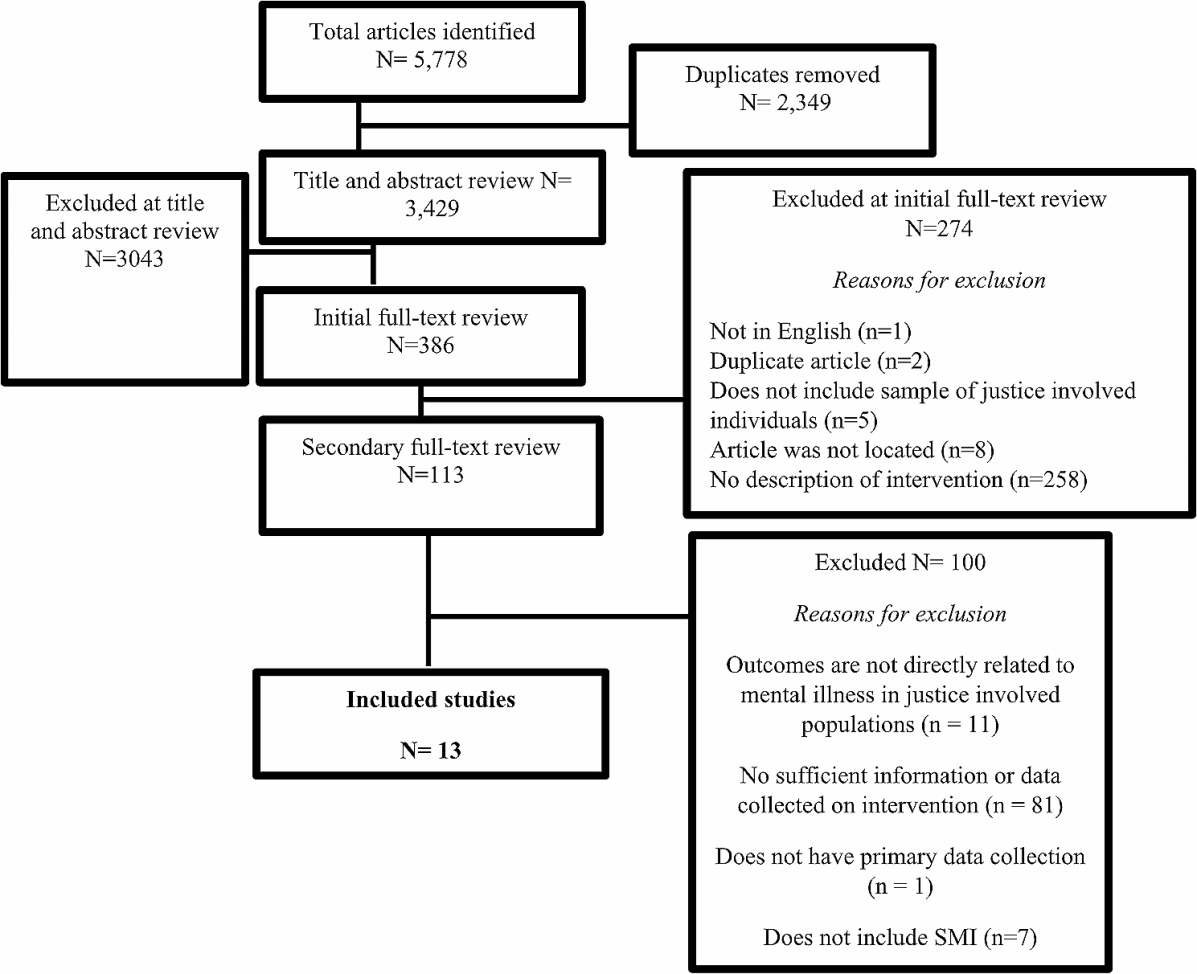
Flow chart of the study

Three of the authors (TB, BWM, MH) reviewed the title and abstracts of 5,778 articles, including the initial 3,429 articles as well as 1,509 articles identified from the updated review conducted in 2021–2022 using exported Excel sheets collated with all articles. Articles were eligible for a full-text review if they met the inclusion criteria described above. Whenever the reviewers could not determine eligibility based on these details in the abstract and the title, the articles moved on to the full-text review phase by default. If the title/abstract was not relevant, then the article was excluded from the review. Any disagreements between reviewers were resolved using consensus procedures. The first and/or second author assisted team members on disagreements that could not be resolved. During this phase, 5,392 articles were excluded. After the title/abstract review phase, eight additional members were added to the coding team to begin reviewing 386 articles in the full-text review phase using Qualtrics as a data management platform. Five pairs of reviewers independently reviewed and coded 20–30 articles (of the 386) that were randomly assigned to them using a random number generator. Reviewers read the full articles more in depth and applied the eligibility criteria described above to determine whether articles should be included in the review. Disagreements at this phase were resolved using consensus procedures where the pair of reviewers met and discussed their agreements and disagreements regarding an article. Wherever reviewers could not resolve the differences after a consensus meeting, a third independent reviewer was invited (e.g., first or second author). During this phase, 273 articles were excluded. The same procedures were followed for the secondary full-text review of 113 potentially eligible articles, where reviewers began coding characteristics of the studies for data extraction. At this phase, 100 articles were excluded for a variety of reasons that are specified in Fig. 1. Once this phase was completed, the review resulted in 13 articles (see Fig. 1: Study Flow Chart) that were found to be eligible for the final data extraction and reporting phase of the study.

### Data collection process

Our team included 10 reviewers with varying levels of mental health research training (i.e., from bachelors to PhD). Reviewers attended and completed multiple trainings focused on systematic review procedures to assure quality of reviews. Grounded on the first and second author’s prior training and best practices from a prior systematic review publication, the training included an overview of the PRISMA guidelines, an introduction to mental health needs of criminal legal involved populations, a review of the systematic review protocol, an orientation of the data extraction form using a practice article, and ongoing team discussions on the coding framework. To facilitate data management, extraction and synthesis, a framework was developed based on the objectives listed in the systematic review protocol. A Qualtrics survey was created based on the framework and was used during the initial and secondary full-test review phase as well as the data extraction. The survey was pilot-tested by a team of 10 reviewers who completed the survey after reviewing the first five articles assigned to them. Feedback from the pilot-review stage was used to enhance the clarity and content of the survey questions. Another practice article was then assigned to assess the consistency of codes and to clarify any misunderstanding of operational definitions. The study team met over Zoom to review responses to the practice article and discuss experiences with the coding process to ensure intercoder agreement for consistency across reviewers. Once procedures were clear and consistent, each reviewer was assigned 20–30 articles to code independently. Weekly team meetings were held throughout the Fall 2020 and Summer 2022 to discuss any issues or concerns regarding the coding process. Pairs of raters then met individually to discuss discrepancies in codes until a consensus was reached. We have achieved 100% concordance between the raters. All articles were independently double-coded.

### Data items

Data extracted for each article included 30 items detailing: (1) reviewer and article information (e.g., publication date, author); (2) intervention details; (3) study design and methods; (4) treatment and/or implementation outcomes; (5) demographics of participants; (6) facilitators and barriers to implementation as conceptualized in the Consolidated Framework for Implementation Research (CFIR); and (7) other comments or concerns regarding the article. Specific items used for the data extraction phase are available in the systematic review protocol (available upon request).

### Risk of bias

We used broad inclusion criteria in order to minimize the risk of publication bias [26]. Thus, studies were included regardless of their outcomes (negative or positive) or research design (i.e., randomized vs. open trials). Appraisal tools from the Equator Network were utilized based on the study designs to enhance quality of reviews [27]. To determine the risk of bias in the design, conduct and reporting of randomized trials, we used the CONSORT checklist, an evidence-based recommendation for reporting randomized trials [28]. To assess the risk of bias in observational studies, we used the Strengthening the Reporting of Observational Studies in Epidemiology (STROBE) checklist [29]. The 32 item Consolidated Criteria for Reporting Qualitative Research (COREQ) checklist was used to determine the risk of bias in qualitative studies (i.e., of implementation barriers and facilitators) [30].

### Data analysis

We used a Qualtrics survey to capture reviewer assessments during the initial and secondary full text reviews and to code eligible articles for data extraction and reporting. A Qualtrics survey was created using our systematic review protocol. The survey was comprised of questions in the areas of names of authors, year of publication, description of the intervention, the study population, the setting, the interventionists, the outcomes reported etc. All reviewers received a link to the Qualtrics survey via email. All articles were assigned to five pairs of reviewers for double coding. Each reviewer completed the Qualtrics survey independently after reading the articles assigned to them, following the data extraction protocol. Once independent reviews were submitted, the second author reviewed the Qualtrics data to identify discrepancies and then notify reviewers to schedule virtual consensus meetings. Once notified via email, pairs of reviewers met to discuss discrepancies in their coding for intercoder agreement. Of note, verbal consensus procedures between coders, such as those detailed in the current review, is considered a best practice approach for improving trustworthiness of qualitative coding [31]. Once consensus was reached for all studies in the full-text review phase, a separate Qualtrics link was used to document the final coding decisions. Data from Qualtrics was exported to SPSS and Microsoft Excel for descriptive analyses of final codes (e.g., frequencies). The review team met to discuss emerging themes and to create a reporting structure based on the objectives of the study. Recurrent themes that were identified were thematically categorized to facilitate reporting.

## Results

A total of 13 eligible articles were included, ranging from publication dates of 1997 to 2020. Designs of the eligible studies included randomized controlled trials (RCTs; *n* = 6), quasi-experimental (*n* = 4), and open-trial (*n* = 3). The types of analysis included were quantitative (*n* = 12) and mixed methods (*n* = 1). Per the World Bank classification of countries [32], all of the eligible studies were from high-income countries. Based on the appropriate appraisal tools from the Equator Network (STROBE for observational studies and CONSORT for RCTs), the quality of the studies were ranked good and above.

### Outcomes reported

All of the studies (*n* = 13) included mental health as their primary outcome (see Table 1). Reduction of symptoms of serious mental illness was reported by the majority of the studies 85% (*n* = 11 studies). Six (46%) of the 13 studies reported both mental health treatment outcomes (reduction in symptoms, improvement in functioning etc.) and implementation outcomes including sustainment of gains (meaning continued delivery of the program or longer-term maintenance of treatment outcomes), feasibility and acceptability, and/or cost-effectiveness. One study reported health services outcomes related to reduction in caseloads, improvement in referrals and triage assessments [33]. Table 1, below presents the reported outcomes in each of the included 13 studies.

**Table 1:** Summary of outcomes reported in the literature

Article	Mental health	Implementation	Medical	Substance use	Recidivism	Other
Broner et al., 2005	X			X	X	
Clayton et al., 2013	X			X	X	
Condelli et al., 1997	X					
Johnson et al. 2008	X	X				Social support
Johnson et al., 2012	X			X		
Johnson et al., 2019	X	X				
Johnson et al., 2020	X	X				
Kamath et al., 2010	X	X	X			
Leidenfrost et al., 2017	X				X	Partner violence
Pillai et al., 2016	X					Employment services
Qiu et al., 2016	X					
Smelson et al., 2019	X	X		X	X	Partner violence
Steadman et al., 2011	X	X			X	

#### Level of evidence

We ranked the strength of evidence based on the strength of evidence pyramid [34]. Accordingly, RCTs were assigned level I, cohort studies or quasi-experimental studies with a comparison group as level II, and open trials and quasi-experimental design with no comparison group were ranked as level III. Table 2 presents the level of evidence and the sample sizes of the 13 studies. A description of changes in mental health outcomes in the 13 studies are also presented in Table 2; significant outcomes are noted when appropriate.

**Table 2:** Studies reporting mental health treatment outcomes

Article	Interventionists	Setting	Sample Size (N)	Study Design	Evidence Strength	Outcomes measured*
Broner et al., 2005	Mental health clinicians and case managers	Community	175	Quasi-experimental (with comparison group)	Level II	*Time spent in prison *Time spent in community *Residential or outpatient treatment *Drug and alcohol use Likelihood of receiving community treatment Life satisfaction Risk of violence
Clayton et al., 2013	Peers	Community	114	Randomized controlled trial (RCT)	Level I	*Psychiatric symptoms *Quality of life *Drug and alcohol use over time *Satisfaction with work Increase in amount of activity Anxiety Depression
Condelli et al., 1997	Mental health clinicians	Correctional facility	209	Open trial	Level III	Serious problem behaviors (suicide attempts, serious infractions, very serious infractions) Correctional restrictions (privileges, keep lock status, discipline) *Mental health crisis service use (emergency medicine and mental health observations) Medication utilization
Johnson et al. 2008	Mental health clinicians	Correctional facility	25	Open trial	Level III	*Presence of depressive symptoms *Severity of depressive symptoms *Perceived social support
Johnson et al., 2012	Mental health clinicians	Correctional facility	38	Randomized controlled trial (RCT)	Level I	*Depressive symptoms Substance use relapse after release
Johnson et al., 2019	Mental health clinicians and nonspecialist counselors	Correctional facility	181	Randomized controlled trial (RCT)	Level I	*Depressive symptoms *Rates of MDD remission *PTSD symptoms *Hopelessness Loneliness Generalized anxiety symptoms Aggression and victimization Social support Suicide ideation
Johnson et al., 2020	Mental health clinicians and nonspecialist counselors	Correctional facility	71	Randomized controlled trial (RCT)	Level I	*Feasibility *Acceptability *Client satisfaction Organizational Readiness *Prison and provider attitudes toward evidence-based practices Provider/Administrator competencies
Kamath et al., 2010	Mental health clinicians	Correctional facility	40	Open trial	Level III	*Bipolar Disorder symptoms *Symptom severity *Quality of life Utilization of antipsychotics *Utilization of antidepressants *Utilization of anticonvulsant
Leidenfrost et al., 2017	Mental health clinicians	Correctional facility	146	Open trial	Level III	*Psychiatric symptoms *Recall *Motivation *Cognitive ability
Pillai et al., 2016	Mental health clinicians and correctional health staff	Correctional facility	19,349	Quasi-experimental (with historical control)	Level II	Rates of referral Rates of triage assessment *Accepted caseloads
Qiu et al., 2016	Mental health clinicians and art therapists	Correctional facility	247	Randomized controlled trial (RCT)	Level I	Depressive symptoms *Negative psychiatric symptoms Anxiety *Anger *Medication adherence *Compliance with rules *Socialization with peers *Sleeping patterns
Smelson et al., 2019	Case managers and peers	Correctional facility	86	Open trial	Level III	*Nights spent in jail *Alcohol use *Drug use *Full time employment Type of Mental health treatment
Steadman et al., 2011	Case managers	Community	447	Quasi-experimental (with comparison group)	Level II	Substance use *Diagnosis of depression *Mental health treatment Medical treatment *New arrests *County jail and state prison incarceration days

*Significant findings for this outcome (between conditions if randomized or quasi-experimental study, pre- post- if an open trial

We also reviewed the implementation outcomes reported across studies in the domains of efficacy, feasibility and acceptability, program effectiveness, cost-effectiveness and maintaining intervention gains. All studies that included implementation outcomes (*n* = 6) focused primarily on the feasibility and acceptability of interventions [33, 35–39]. One study focused on the cost-effectiveness of the implementation of interpersonal psychotherapy for major depressive disorder in a prison population [40].

### The interventionists

Table 2 includes the types of interventionists used in each study. All except three interventions were delivered by mental health clinicians (*n* = 10). Some of the studies reported intervention that were delivered by clinicians in collaboration with case managers [41], correctional staff [33] and art therapists [42]. Only three studies [35, 43, 44] did not involve mental health clinicians.

### The intervention setting

Table 2 also includes the types of settings each intervention took place. Ten of the 13 studies reported that the interventions were delivered inside a correctional facility (jail or prison). The other three studies used interventions delivered in the community setting [41, 43, 44].

### The interventions and intervention characteristics

There was a great variation in types of interventions reported in the included studies. These include clinician-delivered individual or group psychotherapy [39, 40, 45, 46]; group-based module curriculum with components on discharge planning and release safety planning and coping, courtroom behavior, treatment compliance, mental health and substance abuse, anger management and conflict, effective communication skills [37]; adaptation of evidence-based treatment algorithms to improve clinical outcomes [36]; citizenship project including peer support, citizenship classes [43]; intermediate care programs to ease transitions from prison to the community [47]; a new model of care for broadened triggers for mental health referral [33]; wraparound case management and peer support services [35]; art brut therapy [42]; jail and court based diversion programs [41]; and mental health court [44]. All of these interventions were delivered at intercepts 3 and 4 (jail/court-based and during community reentry) in the sequential intercept model (see Table 3) [48]. None of the interventions addressed mental health concerns at earlier or later intercepts.

**Table 3:** Frequencies and proportions of the intervention modalities

Article	Intervention	Modality	Intervention Description	Intercept	Number of studies	% of overall studies
Kamath et al., 2010 Leidenfrost et al., 2017	The Texas Implementation of Medication Algorithms (TIMA) - for bipolar disorder Group based module curriculum	**Cognitive behavioral**	These interventions focus on replacing or modifying behaviors or thought patterns. TIMA is an evidence-based practice guideline for medication treatment of bipolar disorder, major depressive disorder, and schizophrenia in the public mental health system of Texas. Group based module curriculum are interventions that help justice involved individuals acquire life skills, coping mechanisms, anger management skills through various group based educational activities.	Intercept 3: jails and courts	2	15%
Clayton et al., 2013 Condelli et al., 1997 Pillai et al., 2016 Smelson et al., 2019	The Citizenship Project Intermediate care programs Prison model of care Mission CJ	**Systems of care**	The Citizenship Project was designed to address the specific community and social inclusion needs of persons with serious mental illness (SMI) and criminal justice histories by linking them to citizenship-oriented community-based treatment. Intermediate care programs include dedicated staff of mental health and corrections professionals (e.g., clinical psychologist, social worker, occupational/recreational therapist, senior corrections counselor, corrections counselor) offering milieu therapy, individual and group therapy, recreation therapy, task and skills training, educational and vocational instruction, and crisis intervention. Prison model of care includes five steps of screening, referral, assessment, treatment and release planning to improve the consistency and quality of prison mental health in-reach care. Mission CJ combines evidence-based services into a multicomponent system of care that includes critical time intervention, empowering pro social change sessions, dual recovery therapy, peer support, vocational and educational support, and trauma informed care.	Intercept 4: community reentry	4	31%
Johnson et al. 2008 Johnson et al., 2012 Johnson et al., 2019 Johnson et al., 2020	Interpersonal psychotherapy (IPT) for major depressive disorder (MDD) among prisoners	**Interpersonal focus**	IPT is a combination of group-based and individual interpersonal psychotherapy sessions to address conflict in interpersonal relationships and reduce depressive symptoms.	Intercept 3: jails and courts	4	31%
Qiu et al., 2016	Go Beyond Schizophrenia (BGTS) – art therapy program	**Expressive therapy**	Go Beyond the Schizophrenia (GBTS) is an art therapy program that was culturally tailored to Chinese inmates to help with schizophrenia express their emotions through painting and drawing without any limitations and to encourage their creative self-expression, communication of and insight into their concerns.	Intercept 3: jails and courts	1	8%
Broner et al., 2005 Steadman et al., 2011	NYC- Link Diversion Program Mental Health Court	**Court based interventions**	NYC- Link Diverse Program is a citywide program that includes both jail reentry and jail and court-based diversion for those with mental illness entering the courts or any of its 16 jail facilities. Mental Health Courts are post jail diversion programs with the goal of moving persons with serious mental illness out of the criminal justice system and into community treatment without sacrificing public safety based on court sanctions.	Intercept 3: jails and courts	2	15%

To synthesize interventions that were targeting individuals with SMI who have criminal legal involvement, a team of coders followed a thematic analysis to group interventions by categories. Interventions were grouped into five categories based on the modality, focus, and context of the interventions. These categories for modality included cognitive-behavioral, community-based, interpersonal, expressive therapy, and court-based interventions. Table 3 reports the interventions, categories for modality, description of each category, their frequencies across included studies. Due to methodological heterogeneity, not all studies reported effect sizes. However, for those studies that reported effect sizes, the effect sizes ranged from (*p* < 0.001) [36] to (*p* < 0.0001) [37].

### Length of the intervention and the problems addressed

The length of intervention delivery varied across the studies ranging from 2 months to over a year. Only three of the studies described an intervention delivered for over a year [33, 35, 44]. Additional three studies reported interventions delivered for 7–12 months [41–43]. The rest of the studies reported the use of an intervention that was delivered for six months or under.

Presenting problems of participants across all articles were synthesized by each of the following categories: mental health, medical, substance use, or recidivism. All 13 studies reported mental health as a primary outcome. Additional outcomes reported include recidivism (*n* = 5, 38%), substance use (*n* = 4, 31%), and medical care (*n* = 1, 8%). Three of the articles included another option describing studies with a focus on additional presenting problems related to intimate partner violence, violence towards inmates, employment service utilization, and changes in social support. Of note, these summarized presenting problems were not mutually exclusive, with 54% of articles (*n* = 7) attempting to address multiple presenting problems. See Table 1 for information on all outcomes measured.

### Barriers and facilitators to the implementation of interventions reported in the literature

#### Barriers

Of the 13 articles included in this systematic review, 31% (*n* = 4) reported at least one barrier to implementing the intervention in criminal justice (*n* = 2) or community-based rehabilitation settings (*n* = 2). The reported barriers included lack of external policy and incentives (e.g., correctional/administrative policy, clinical culture in the correctional institutions [36, 46], patient needs and resources, design quality and packaging, personal attributes of the study participants, implementation climate, and available resources. See Table 4. Two studies reported barriers related to the inner and outer setting of interventions. Other studies reported barriers relating to the intervention process, characteristics of the individuals in the interventions, or a barrier that did not fall within the CFIR constructs (e.g., high staff turnover). Of note, there were no barriers reported related to the intervention characteristics.

**Table 4:** Barriers reported in the sample according to the CFIR constructs

Barrier	n	%
No Barriers Reported	9	69%
**Outer Setting**	2	15%
*External Policy & Incentives*	1	8%
*Patient Needs & Resources*	1	8%
**Characteristics of Individuals**	2	15%
*Other Personal Attributes*	2	15%
**Inner Setting**	3	23%
*Implementation Climate*	1	8%
*Available Resources*	1	8%
*Readiness for Implementation*	1	8%
**Other**	1	8%
*High Staff Turnover*	1	8%

#### Facilitators

Four articles (30%) reported at least one facilitator for intervention implementation (Table 5) in criminal justice (*n* = 3) or community-based rehabilitation settings (*n* = 1). Guided by the CFIR framework, of the four studies that reported facilitators, one of them reported facilitators related to the intervention characteristics which included design quality and packaging, evidence strength and quality, cost, relative advantage, and adaptability. Three of the studies reported facilitators relating to the inner setting of the intervention, which included implementation climate [41], access to knowledge and information [41, 46], available resources [43], and relative priority [46]. Five specific facilitators were reported more than once across studies, including implementation climate, knowledge and beliefs about the intervention, patient needs and resources, evidence strength and quality, and cost. Facilitators that were reported only once were related to intervention execution, personal attributes of the intervention participants, access to knowledge and information, available resources, design quality, relative advantage, and adaptability of the intervention.

**Table 5:** Facilitators reported in the sample according to the CFIR constructs

Facilitator	n	%
**No Facilitators Reported**	9	69%
**Outer Setting**	1	8%
*Patient Needs & Resources*	1	8%
**Characteristics of Individuals**	3	23%
*Knowledge & Beliefs about the Intervention*	2	15%
*Other Personal Attributes*	1	8%
**Inner Setting**	3	23%
*Access to Knowledge & Information*	1	8%
*Available Resources*	1	8%
*Relative Priority*	1	8%
*Implementation Climate*	1	8%
**Intervention Characteristics**	2	15%
*Design Quality & Packaging*	1	6%
*Cost*	1	8%

## Discussion

The goal of the current paper was to systematically review the existing research on mental health interventions for individuals with serious mental illness who have criminal legal involvement. Given that the criminal legal system has become the country’s largest *de facto* provider of mental health services [8], there is a critical need to understand the effectiveness and implementation potential of evidence-based mental health interventions on individual mental health outcomes in these settings. Our review identified 13 studies that were found eligible for data extraction and reporting. Findings from synthesizing these literatures present mixed results, with some studies supporting the clinical utility and implementation potential of delivering interventions for individuals with SMI and criminal legal involvement, but others pointing to gaps in the extant literature and specific avenues for future research.

Of note, articles that were found eligible for this systematic review were somewhat nascent, beginning with a study developed in 1997. Additionally, there was significant heterogeneity among the research included in this review. Studies utilized a wide range of trial designs (e.g. RCTs, quasi-experimental, open trial) and focused on various mental health disorders as the primary outcome of interest. Across studies, however, all findings showed indications of positive influences on primary mental health outcomes, including improvement in depression, bipolar disorder, and PTSD symptoms. This should be interpreted with caution, as studies ranged in design and approach to measurement. Given that slightly less than 1% of individuals in America are currently incarcerated [49] and around 40% of those incarcerated in prisons have a mental health diagnosis [50], the consistency of these findings point to dissemination of interventions in criminal legal settings as an important tool for improving public health.

Interestingly, 11 of the studies found in our literature search had to be excluded as they did not include mental health as the primary outcome of a mental health intervention. In our review, IPT interventions constitute nearly a third of all interventions and were evaluated by a single investigator. Moreover, the majority of studies also reported a variety of secondary criminal legal, health services, and functional and behavioral health outcomes (e.g., recidivism, substance use, and engagement in medical care). While these studies largely found promising directions of the intervention’s influence for these other outcomes, the frequency of including outcomes that are adjacent to, but not directly related to the intervention, suggests a possible bias in the literature to focus on outcomes beneficial to society and less so for the population under study. This targeting of multiple problems and emphasis on multi-level approaches is important given that mental health concerns for criminal legal-involved populations is multifaceted and often involve multiple systems of care [19]. Nonetheless, our review suggests that research on the impacts of mental health interventions for people with SMI involved in some aspect of the criminal justice system could benefit from increased focus on mental health itself.

Results also point to the need for innovations around intervention delivery and the need for adopting interventions with strong evidence for improving mental health outcomes. Additionally, the majority of studies utilized mental health clinicians to deliver interventions; only three examined the efficacy of services delivered by non-clinical staff (in all of these cases, interventions were delivered by case workers). While this reflects long-standing divisions between professional roles, a growing literature points to the implementation potential of utilizing non-specialists to deliver interventions. For instance, a recent commentary suggests that individuals with lived experience with mental and behavioral health disorders and/or involvement in the criminal legal system, may be uniquely suited to support services for incarcerated individuals [51]. Peer-delivered interventions may be less stigmatizing [52] feasible within correctional settings [53], and have the potential to be cost-effective in resource limited contexts [54] including criminal legal contexts. An emerging body of literature also suggests that peers can deliver interventions with fidelity [55]. Given limitations to the reach and availability of specialist care relative to the mental and behavioral health needs of individuals in criminal legal settings, it will be important to explore alternative delivery approaches and workforces to increase access to Interventions, reduce mental health stigma and improve social norms around treatment engagement [56]. Moreover, the structural (e.g., restrictive prison policies that do not allow individuals with felony to be around others with criminal records) and individual (i.e., fear of stigma, relieving the trauma of incarceration) challenges of engaging formerly incarcerated individuals with SMI must be acknowledged.

The Sequential Intercept Model (SIM) maps points of intersection between individuals with mental and behavioral health needs and the larger criminal legal systems [22, 48]. While the reentry and community corrections periods correspond to the final two points of intercept, the SIM also identifies four earlier points of intersection that may offer opportunities to deliver Interventions. For instance, none of the interventions reviewed above offer services when individuals first interact with local law enforcement (at the point of arrest) or during initial court hearings and detention, despite some evidence suggesting that individuals are at increased risk of suicide immediately after arrest and initial detainment [57, 58]. Interventions at earlier intercepts are fewer likely due to the fact that a vast majority of individuals with SMI are unstably housed, hence, making it difficult to conduct multi-session interventions [59]. Moreover, shared sets of barriers were experienced across the interventions regardless of the intercepts targeted. This in part could be attributable to the fact that the criminal legal system is not set up to effectively respond to mental health care needs [60, 61].

In this review, the types of interventions and the outcomes of interest seem to have varied along the lines of the study settings. Although mental health outcomes were the primary focus of the included studies, five of the studies evaluated criminal legal outcomes such as recidivism in addition to the mental health outcomes [35, 37, 41, 43, 44]. Another study evaluated the effect of an intervention on reducing serious problem behaviors within a correctional facility [42]. Studies focusing on the transition from correctional setting to the community targeted improvement in clinical outcomes, which in turn contribute to better service linkages, easier transitions and improvement in criminal legal outcomes.

The current review also focused on understanding the barriers and facilitators to implementing mental health interventions with individuals who have SMI and in criminal legal settings. It’s important to note that implementation of mental health interventions in criminal legal settings can be fraught with inner and outer context challenges related to low resources, lack of proper staffing and/or training, intervention characteristics misaligned with treatment needs, among others. These settings may also be highly controlled and restrictive, which may affect the extent to which interventions are adapted or tailored to be culturally responsive for individual needs [13, 46]. Research guiding implementation of evidence-based mental health interventions in criminal legal settings is limited and far behind by comparison of work done in health systems. Yet, implementation is critical for accelerating uptake and maximizing sustainable, positive outcomes of mental health interventions. In an effort to understand the barriers and facilitators to implementing these interventions within criminal legal settings, the current review applied the CFIR framework to identify and categorize aspects of implementation across studies. Results highlight various elements related to implementation that can be used to further tailor implementation strategies to an organization’s context. Specifically, findings point to the inner setting and intervention characteristics as the most commonly cited facilitators, implicating the need to focus on these aspects during treatment adaptation to ensure an appropriate fit to the treatment context. Interestingly, however, no specific barriers or facilitators were identified by more than two studies. This indicates that a majority of studies did not report on implementation strategies, factors, or outcomes. This highlights the need for a standardized and consistent reporting of barriers and facilitators encountered during the implementation of interventions in these settings to inform future efforts. Additionally, only one study examined the cost-effectiveness of these approaches [40], underscoring the need for a robust design evaluating the costs associated with implementing mental health interventions in criminal legal settings.

Further, studies included in this review were exclusively from criminal legal settings in high-income countries. While many low- and middle-income countries (LMICs) have small prison populations, a number of risk factors inherent to these settings (including overcrowding, lack of resources, etc.) create further barriers to accessing mental health services [62]. Moreover, incarceration rates in low-and middle-income countries have been increasing [12]. Given that we found no intervention studies conducted in LMICs, there is a compelling need to examine the comparative efficacy and implementation potential of interventions in the U.S. to evaluate whether these approaches may meet the growing need for services within these criminal legal systems.

### Limitations

While the reported results carry important implications for mental health in the criminal legal system, there are also some limitations worth stating. Our inclusion criteria focused on studies that are peer-reviewed, excluding other grey literature and unpublished reports, presenting a potential publication bias. Moreover, we were unable to find studies from LMICs that met our inclusion criteria. Therefore, the evidence must be interpreted with caution. Intervention studies focusing on serious mental illness in the criminal legal system in LMICs are needed.

## Conclusions

Despite these gaps in the extant literature, this review provides support for both disseminating and implementing interventions for individuals with SMI who have criminal legal involvement. While future research is needed to examine how interventions could be delivered utilizing different workforces, at different points of intersection with the criminal legal system, and in other settings, results broadly highlight the promising implications of interventions for individuals with SMI who are criminal legal-involved. In turn, increasing access to evidence-based approaches has the potential to improve outcomes, disrupt cycles of reincarceration, and reduce the disproportionate burden of mental health disorders within the criminal legal system. More RCTs or other studies with fully powered samples, however, are needed to determine effectiveness in mitigating negative mental health outcomes for these populations.

### Electronic supplementary material

Below is the link to the electronic supplementary material.

Supplementary Material 1

Supplementary Material 2

## Data Availability

Systematic review protocol is available from the first author upon request.

## Abbreviations

CFIR: Consolidated Framework for Implementation Research
CONSORT: Consolidated Standards of Reporting Trials
COREQ: Consolidated Criteria for Reporting Qualitative Research
EBI: Evidence-based interventions
HIC: High-income countries
IPT: Interpersonal Psychotherapy
LMICs: Low- and middle-income countries
PRISMA: Preferred Reporting Items for Systematic Reviews and Meta-Analyses
PTSD: Post-traumatic stress disorder
RCT: Randomized controlled trial
STROBE: Strengthening the Reporting of Observational Studies in Epidemiology
SIM: Sequential Intercept Model
SMI: Serious mental illness
