# Supplementary material for: Mental health interventions for individuals with serious mental illness in the criminal legal system: a systematic review
Source: BMC Psychiatry. 2024 Mar 12;24:199. doi: 10.1186/s12888-024-05612-7 (PMC10935949; doi:10.1186/s12888-024-05612-7)
Supplement: Supplementary file 2 — Supplementary Material 2 [file 12888_2024_5612_MOESM2_ESM.docx]

Supplementary file 2: Limitations of included studies

| **Authors** | **Quality Assessments** |
| --- | --- |
| Broner et al., 2005 | - Small sample size and potential sample bias - The possibility of Type I error, requiring caution in interpreting results and necessitating future study and replication. - Possible bias since programs did not consistently record all information (date of referral, program acceptance, whether the diversion failed prior to jail release), - Lengths of stay in jail were calculated from entrance into the correctional system |
| Clayton et al., 2013 | - The extra assistance and attention intervention participants received may have facilitated the outcomes reported regardless of the intervention received. - Study design did not allow to differentiate the relative importance of peer mentor, class, and valued role components in producing the findings. - The development of the three-pronged intervention itself— peer mentor support, classes, and valued roles represented the investigators’ ‘‘reasonable’’ approach to translating our theoretical framework of the five Rs of citizenship (rights, responsibilities, roles, resources, and relationships) into program elements. |
| Condelli et al., 1997 | - Stringent exclusion criteria that excluded some inmates who were too unwell or who recovered too quickly. |
| Johnson et al. 2008 | - Small sample size (n = 26) - Lack of a control group, the lack of formal treatment adherence or competence monitoring, use of self-report measures of substance use only, and assessments after the acute treatment phase only. Participants received other services and almost half of the participants were on antidepressants, making it impossible to conclude that the observed changes were due to IPT - Study does not provide any information about whether the women were able to maintain their gains in depressive symptoms and social support after they left prison or whether these gains translated into lower substance use disorder relapse risk postrelease. |
| Johnson et al., 2012 | - Small sample size, short follow-up period limiting ability to meaningfully test other potentially important outcomes (e.g., prison suicide attempts, recidivism). - It is possible that non-specific factors may have accounted for the decrease in depressive symptoms during incarceration for IPT condition compared to the matched attention placebo condition. |
| Johnson et al., 2019 | - The 3-year project period and group treatment design did not allow for study counselors to have training cases, necessitating secondary analysis with the subset of the sample randomized in waves in which counselors had run at least one previous IPT group. - In addition, unexpectedly low rates of self-reported suicide ideation and of discipline/incident reports and time spent in isolation in the sample reduced power for these outcomes. - The tight project period and short sentence lengths in the states did not allow for follow-up beyond 3 months post-treatment. - Study did not address postrelease effects of treatment. |
| Johnson et al., 2020 | - Concerns of generalizability. - The provider/administrative surveys did not compare answers of the 8 survey respondents involved in delivering IPT to other survey respondents or compare characteristics of those who did or did not respond to the survey. - Internal consistency reliability on 4 of the 13 CAI subscales is “fair” (0.52–0.60), but this has minimal or no implications for our conclusions. |
| Kamath et al., 2010 | - Small sample of 20 M and 20 F, qualitative study is descriptive with issues on generalizability to other cases, no control for the intervention environment. |
| Leidenfrost et al., 2017 | - Since the participants were all recruited from a treatment unit within a correctional setting and placement on the unit is considered, by some, more desirable to other parts of the jail, this may have biased reports of group usefulness in the belief that it would allow them to remain on the unit. - Varying time period may have impacted the recall ability of some participants. - Varying effects due to the use of psychiatric medications and pharmacodynamic drug processes were not analyzed. |
| Pillai et al., 2016 | - Naturalistic study design, not all variables impacting on outcomes were manipulated. - The data collected relied on clinical file information collected for purposes other than research. Data collection for the post-PMOC period also commenced from the day of introduction, without giving the new ways of working time to become established. - Likely impacted the effect size of the intervention. |
| Qiu et al., 2016 | - Concerns of generalizability (sample is from mainland China) - Effects of the intervention could be explained by many potential variables although statistically significant. - Potential confirmation bias- inmates were more open and less defensive when they were in a focus group, and they communicated much better orally than in written formats. |
| Smelson et al., 2019 | - Limitations due to a pre-test/post-test design including lack of a comparison group and the fact that over time, clients might have improved irrespective of the intervention delivery. - Short follow up period (6 months), effect of the intervention on the outcomes post 6 months is unknown. - Service providers did not consistently have available space within the court to deliver MISSION-CJ services nor did they consistently track the contacts delivered inside the court setting versus in the community. - Reliance on self-reported data, such as jail days and re-arrest, which are best captured in rigorous methodically driven studies by official record data. |
| Steadman et al., 2011 | - Data did not comprehensively address the key questions of who the courts are most effective for or what mechanisms produce positive outcomes. |
